# Evaluation of the Antioxidant, Anti-Inflammatory, and Antimicrobial Activity of a *Cannabis sativa*-Infused African Product Used for Wound Healing

**DOI:** 10.3390/ijms27156815

**Published:** 2026-07-29

**Authors:** Siboniso Sithole, Xolani Shezi, Mlondolo Mavundla, Carl Mateta, Sphamandla Hlatshwayo, Sanele Mhlungu, Nokukhanya Thembane, Phumzile Afrika, Sibusiso Senzani, Exnevia Gomo, Nceba Gqaleni, Mlungisi Ngcobo

**Affiliations:** 1 Sub-Discipline of Traditional Medicine, School of Medicine, College of Health Sciences, University of KwaZulu-Natal, Durban 4041, South Africa; mavundlam@ukzn.ac.za (M.M.); 210505551@stu.ukzn.ac.za (S.H.); 209504114@stu.ukzn.ac.za (S.M.); 215068694@stu.ukzn.ac.za (N.T.); 209507332@stu.ukzn.ac.za (P.A.); gqalenin@ukzn.ac.za (N.G.); 2 Independent Researcher, Richmond 3780, South Africa; xolanisheziii12345678@gmail.com; 3 National Institute of Health Research, Ministry of Health and Child Care, Harare P.O. Box CY 1122, Zimbabwe; carlmateta@gmail.com; 4 Department of Biomedical Sciences, Faculty of Applied and Health Sciences, Mangosuthu University of Technology, Durban 4026, South Africa; 5 Discipline of Medical Microbiology, School of Medicine, College of Health Sciences, University of KwaZulu-Natal, Durban 4000, South Africa; senzanis@ukzn.ac.za; 6 Medical Laboratory Sciences Unit, Faculty of Medicine and Health Sciences, University of Zimbabwe, Mount Pleasant, Harare P.O. Box MP 167, Zimbabwe; exgomo@gmail.com; 7 Africa Health Research Institute, 3rd Floor K-RITH Tower Building, Nelson R. Mandela School of Medicine, Durban 4001, South Africa

**Keywords:** wound healing, African traditional medicine, anti-inflammation, antioxidant, antimicrobial, KwaZulu-Natal

## Abstract

Product Shezi (PS) is a polyherbal African traditional medicine (ATM) formulated from six known South African medicinal plants and is used for treating cutaneous wounds. However, its ethnopharmacological properties have not been scientifically validated. This study aimed to evaluate the antioxidant, anti-inflammatory, and antimicrobial activities of PS in vitro. Aqueous and methanolic extracts were prepared and qualitatively screened for phytochemical constituents. Cytotoxicity in fibroblasts and macrophages was assessed using an ATP-based viability assay. Antioxidant activity was evaluated using the 2,2-diphenyl-1-picrylhydrazyl (DPPH) radical scavenging and a hydrogen peroxide (H_2_O_2_)-induced oxidative stress model. Anti-inflammatory effects in lipopolysaccharide (LPS)-stimulated macrophages were measured using the Griess reagent system, a human prostaglandin E_2_ (PGE_2_) ELISA, and a bovine serum albumin (BSA) anti-denaturation assay. Antimicrobial activity was assessed by twofold serial broth microdilution, agar well diffusion, and a crystal violet biofilm assay. Phytochemical screening confirmed the presence of saponins, alkaloids, tannins, glycosides, terpenoids, flavonoids, and steroids. PS exhibited IC_10_ values of 3 and 10 μg/mL in fibroblasts and macrophages, respectively. PS (1–5 μg/mL) showed 70% DPPH scavenging potential and potent H_2_O_2_ cytoprotection (*p* < 0.001). Nitric oxide inhibition was non-significant (*p* > 0.05) whilst PGE_2_ decreased significantly (*p* < 0.05) compared to LPS-stimulated cells. BSA denaturation was inhibited in a dose-dependent manner (*p* < 0.05). *Staphylococcus aureus* and *S. epidermidis* were the only microorganisms susceptible to PS (MIC 650–5000 μg/mL) with low SI (SI ≈ 0.05–0.06), while others were resistant. PS showed no antibiofilm activity (crystal-violet assay). Overall, PS demonstrated antioxidant and anti-inflammatory activities and selective antibacterial effects against planktonic bacteria in vitro. However, further optimization of extraction methods, detailed mechanistic studies, and in vivo investigations are warranted to substantiate therapeutic relevance.

## 1. Introduction

Wound healing is a complex reparative process consisting of four overlapping phases: hemostasis, inflammation, proliferation, and remodeling or maturation. The inflammatory phase represents an essential protective response to tissue injury and pathogen invasion. This process is crucial in achieving complete wound healing. The chief goal of this phase in the wound healing process is to purge cellular debris and phagocytose all the pathogens and foreign substances from the wound site [1]. However, the inflammatory phase is susceptible to interruption by both endogenous and exogenous factors, which may delay progression to the proliferative and remodeling stages [2]. The delay in wound healing affects the patient’s quality of life and healthcare systems. A recent study by Holzer-Geissler et al. [3] indicated that prolonged inflammation contributes to the development of chronic wounds. Dysregulated inflammation typically promotes membrane alterations, increases protein denaturation, and results in scar formation [4]. More broadly, chronic inflammation has been implicated in the pathogenesis of multiple disorders, including type 2 diabetes and aging-related conditions [5]. The inflammatory phase in chronic wounds, such as diabetic foot ulcers, lasts longer than that of acute wounds [6].

Precise regulation of inflammation and oxidative stress is a critical factor in complete wound healing. During the inflammatory phase, reactive oxygen species (ROS), such as hydrogen peroxide, hydroxyl radical, and nitric oxide, are produced by inflammatory cells to initiate the wound-healing process [7]. ROS are produced in low concentrations as a host response mechanism to eliminate infections at the wound site [8]. However, excessive ROS production leads to oxidative stress, disrupting membrane integrity. Disproportionate ROS production also impairs lipid and protein homeostasis and damages extracellular matrix components, thereby compromising healing [9]. In the human body, low levels of nitric oxide are essential for enhancing antimicrobial activity, especially after injury; thus, excessive concentrations contribute to prolonged inflammation [10]. Cytokines, as well as other mediators of inflammation such as prostaglandins, are also secreted during an inflammatory response [11]. An increase in pro-inflammatory cytokines, such as IL-1 and TNF-α, on the wound site promotes prostaglandin synthesis [12]. In inflamed tissue, prostaglandin E2 is released in excess, leading to peripheral nociceptor activation [13]. Overproduction of prostaglandins via cyclooxygenase pathways during wound healing results in pain. Therefore, persistent oxidative stress and unresolved inflammation are hallmarks of chronic, non-healing wounds [14]. In general, antioxidants are grouped into natural (enzymic antioxidants) and synthetic antioxidants. Synthetic antioxidant therapies have been developed to combat oxidative stress in cutaneous wounds. It has been reported that synthetic antioxidants are associated with some limitations, including low bioavailability and reduced bioactivity when topically applied directly to wounds [15].

Bacterial infection has been regarded as the major cause of delayed wound healing [16]. In the first week of injury to the dermal tissue, Gram-positive bacteria predominate the wound site. After a week, Gram-negative bacteria then colonize the wound [17]. Many pathogenic bacteria adhere to the wound surface and form biofilm-structured microbial communities embedded within a protective extracellular polymeric matrix. Species such as *Staphylococcus aureus* (*S. aureus*) and *Pseudomonas aeruginosa* (*P. aeruginosa*) are particularly adept at forming biofilms, which enhance their persistence and thus exacerbate infections and compromise wound healing [18]. These infections complicate wound management and further deteriorate clinical outcomes [19]. A recent study has shown a tight relationship between microbial infection and the wound-healing process [20]. Bacterial tolerance to antimicrobial agents at lethal concentrations has been attributed to their presence as a biofilm at the wound site [21]. Emerging evidence suggests that biofilm resistance to antimicrobial agents is 1000 times higher than that of planktonic cells [22]. This is largely due to the antibiotic penetration barrier, which consists of a specialized polysaccharide complex surrounding the biofilm bacteria [23]. Although antibiotic resistance may arise naturally, inappropriate and excessive antibiotic use has accelerated the emergence of multidrug-resistant (MDR) strains, contributing to treatment failure, increased healthcare costs, and heightened morbidity and mortality [24]. The widening gap between antimicrobial development and the rapid evolution of resistance underscores the urgency of identifying alternative therapeutic strategies.

Non-steroidal anti-inflammatory drugs (NSAIDs) such as aspirin, ibuprofen, acetaminophen, and diclofenac are widely used in humans to inhibit inflammation [25]. The efficacy of NSAIDs in modulating inflammation is achieved by inhibiting prostaglandin synthesis and the cyclooxygenase enzyme [26]. Since inflammation is involved in many medical conditions, including wounds, the demand for anti-inflammatory drugs is very high. Antibiotics for wound-related infection may include vancomycin, ceftriaxone, gentamicin, and ciprofloxacin. One limitation of using conventional anti-inflammatory and antimicrobial drugs already on the market is the potential for severe complications and side effects [26]. Side effects may include gastrointestinal ulceration, stroke, hyperglycemia, perforation, and acute renal ulcer after oral administration [27]. Therefore, a search for alternative anti-inflammatory and antimicrobial agents from medicinal plants with reduced unwanted side effects associated with conventional treatments is warranted.

Medicinal plants have gained considerable attention in the field of wound healing in the past decade. Recent studies demonstrated that African traditional medicine (ATM) products possess antioxidant and anti-inflammatory activities [27]. These ATM products have been demonstrated to promote anti-inflammatory activities by inhibiting nitric oxide production, the lipoxygenase enzyme, and prostaglandin synthesis [28]. Their continued relevance is supported by data showing that over 80% of people on the African continent rely on traditional medicine as their primary source of healthcare. This includes treatment of skin-related diseases and associated infections. Globally, there has been increasing interest in studying medicinal plants as alternative treatments for inflammation to reduce limitations of conventional medicines [29]. However, there are limited ethnopharmacological studies on ATM products used in wound treatment. Among ATM products traditionally used for the treatment of wounds in South Africa is Product Shezi (PS), a *Cannabis sativa* (*C. sativa*)-infused polyherbal ATM formulated by a traditional healer in KwaZulu-Natal using five additional known medicinal plants for topical wounds. Product Shezi is a polyherbal African traditional medicine used for the topical treatment of wounds and is applied once daily according to the traditional healer’s instructions. It is formulated from six medicinal plants, with *Cannabis sativa* constituting 50% of the final preparation, while the remaining 50% consists of five known South African medicinal plants, including *Drimia altissima* (L.f.). Ker Gawl., *Albuca fastigiata* Dryand, *Bulbine latifolia* (L.f) Spreng, *Hypoxis hemerocallidea* Fisch., C.A. & Ave-Lall, and *Hypericum aethiopicum* Thumb. The interest in PS is based on anecdotal evidence from the traditional healer regarding its efficacy in treating chronic wounds (Supplementary Figure S1). Therefore, the aim of the study was to evaluate the in vitro antioxidant, anti-inflammatory, and antimicrobial activities of PS.

## 2. Results

### 2.1. Qualitative Phytochemical Analysis

The phytochemical analysis studies indicated the presence of all the prominent phytochemicals in PS (Table 1).

### 2.2. Cytotoxicity Activity

**Figure 1:**
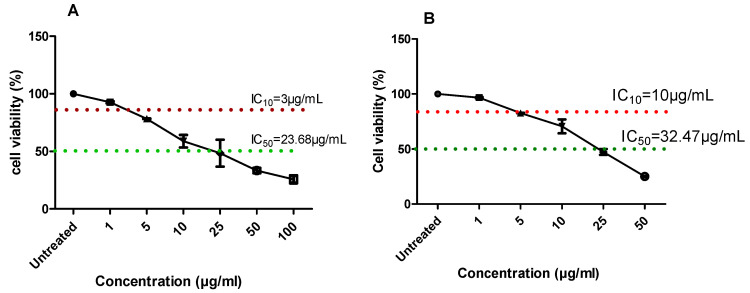
Cytotoxicity effects of PS on the viability of (**A**) fibroblast and (**B**) macrophage cells. The IC_50_ of PS on fibroblast cells was 23.68 µg/mL, with an IC_10_ of 3 µg/mL. The IC_50_ of PS on macrophage cells was 32.7 µg/mL, with an IC_10_ of 10 µg/mL. Data is presented as mean ± SE (*n* = 9). Experiments were conducted in triplicate and repeated in three independent studies.

A dose-dependent reduction in fibroblast and macrophage cell viability was observed following treatment with increasing concentrations of PS, compared to the untreated control. Higher concentrations of PS above 50 µg/mL induced the most pronounced cytotoxic effects in both fibroblast and macrophage cells (Figure 1). The IC_50_ of PS on fibroblast cells was determined to be 23.68 µg/mL (Figure 1), with an IC_10_ value of 3 µg/mL. For macrophage cells, the IC_50_ value was established at 32.47 µg/mL and the IC_10_ at 10 µg/mL (Figure 1B). Concentrations ranging from the IC_10_ and lower were used for further experiments.

### 2.3. Antioxidant Activity

#### 2.3.1. DPPH

**Figure 2:**
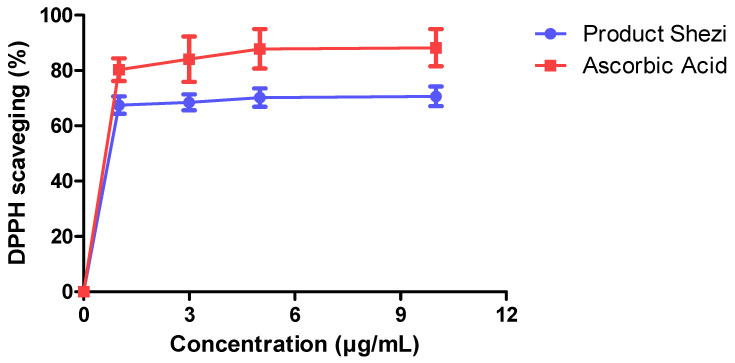
DPPH radical scavenging activity of PS extract. The ATM product scavenged up to 70% of DPPH, with an IC50 of 0.72 µg/mL, while ascorbic acid showed a significantly higher scavenging capacity of up to 94%, with an IC_50_ of 0.21 µg/mL. Data is presented as mean ± SE (*n* = 9). IC_50_ values determined by nonlinear regression analysis. Experiments were conducted in triplicate and repeated in three independent studies.

All tested PS concentrations exhibited antioxidant activity by scavenging DPPH radicals. At concentrations ranging from the IC_10_ of treated fibroblasts, PS was able to scavenge up to 70% of free radicals. The standard antioxidant, ascorbic acid, exhibited a significantly higher scavenging capacity of up to 94% (Figure 2). The IC50 of PS was 0.72 µg/mL, and that of ascorbic acid was 0.21 µg/mL. Experiments were conducted in triplicates and repeated in three independent studies.

#### 2.3.2. Cytoprotective Effect of Product Shezi Against Hydrogen Peroxide

**Figure 3:**
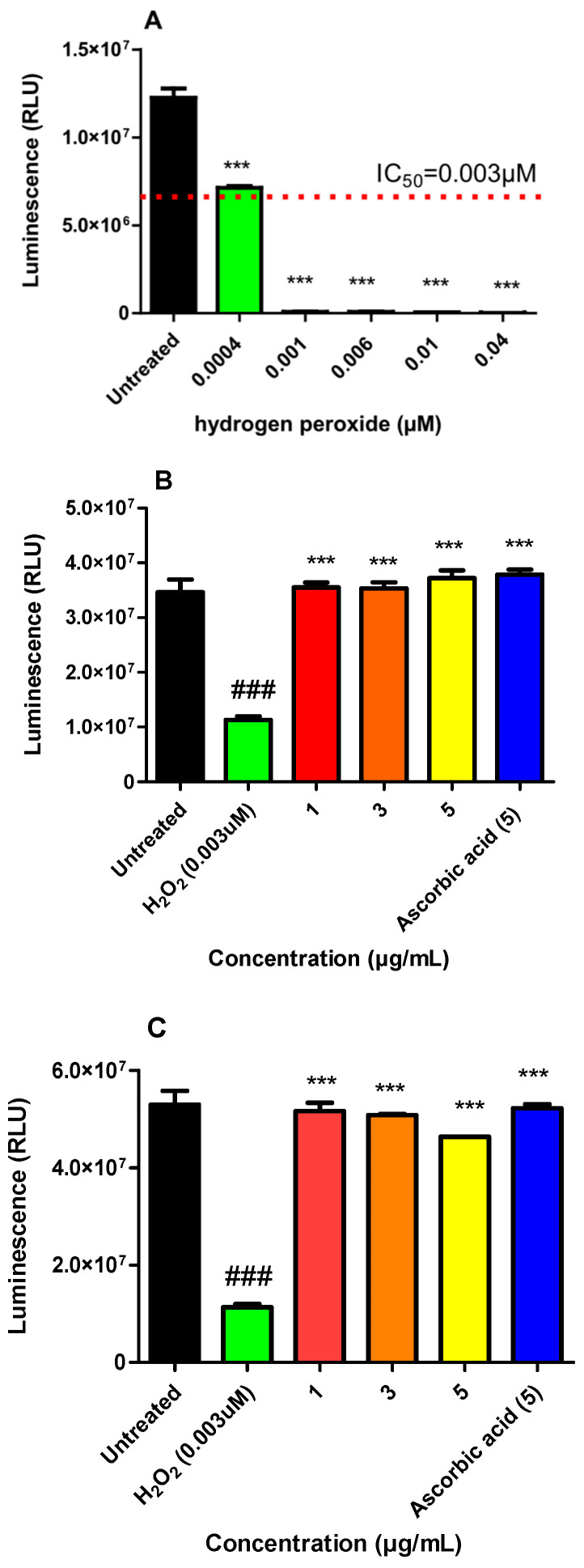
Viability of fibroblast cells treated with H_2_O_2_ only after 3 h of treatment (**A**), simultaneous treatment with H_2_O_2_ and PS (**B**), and after pre-treatment with PS for 24 h, then treated with H_2_O_2_ (**C**). PS significantly protected fibroblasts against H_2_O_2_-induced cytotoxicity in both strategies. Data is presented as mean ± SE (*n* = 9). Statistical analyses were conducted using one-way analysis of variance (ANOVA) to evaluate differences among groups. When significant differences were detected, Tukey’s post hoc multiple comparison test was applied to identify pairwise differences between group means. Statistical analyses: ^###^ *p* < 0.001 compared with the sample of untreated control, and *** *p* < 0.001 compared with H_2_O_2_. Experiments were conducted in triplicate and repeated in three independent studies.

Fibroblast cells were initially treated with increasing concentrations (0–0.0004 µM) of hydrogen peroxide (H_2_O_2_) to model the induction of oxidative stress. A concentration-dependent response was observed after 3 h of exposure, with higher H_2_O_2_ concentrations resulting in greater cellular stress (Figure 3A). Based on these results, a dose of 0.003 µM for H_2_O_2_ was selected for subsequent experiments to evaluate the cytoprotective effect of PS. In the simultaneous treatment strategy, PS significantly (*p* < 0.001) reduced H_2_O_2_ cytotoxicity over 3 h of treatment (Figure 3B). Notably, complete protection was observed at a concentration above 3 µg/mL in the pretreatment model. Furthermore, when cells were pretreated with PS for 24 h prior to H_2_O_2_ exposure, concentrations ranging from 1 to 5 µg/mL effectively protected (*p* < 0.001) fibroblast cells from oxidative stress. PS exhibited cytoprotective effects in both strategies comparable to those of ascorbic acid, a well-known antioxidant.

### 2.4. Anti-Inflammation

#### 2.4.1. Inhibition of Nitric Oxide

**Figure 4:**
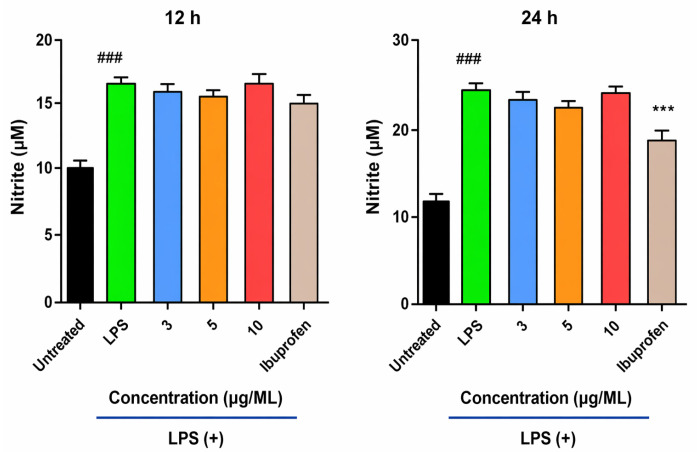
Effect of PS on nitric oxide production in LPS-stimulated macrophages after 12 and 24 h. PS did not significantly inhibit nitric oxide production after 12 and 24 h of treatment when compared to LPS-stimulated macrophages. Statistical analyses were performed using one-way ANOVA followed by Tukey’s post hoc multiple comparison test to compare all pairwise group means. Results are presented as mean ± standard deviation (SD), and differences were considered statistically significant at *p* < 0.05. Statistical analyses: ^###^ *p* < 0.001 compared with the untreated sample, and *** *p* < 0.001 compared with LPS. Experiments were conducted in triplicate and repeated in three independent studies.

Lipopolysaccharide (LPS) was used to induce an inflammatory response in macrophages. The results showed that cells treated with LPS exhibited a significant (*p* < 0.001) increase in nitric oxide production compared to unstimulated cells (Figure 4). The inhibition of overproduction of nitric oxide was observed when LPS-stimulated macrophages were treated with PS concentrations after 12 and 24 h, although the observed effect was not significant *p* > 0.05. In contrast, a significant reduction in nitric oxide levels was only observed after 24 h of treatment with ibuprofen, a standard anti-inflammatory drug (Figure 4).

#### 2.4.2. Inhibition of Prostaglandin Production

**Figure 5:**
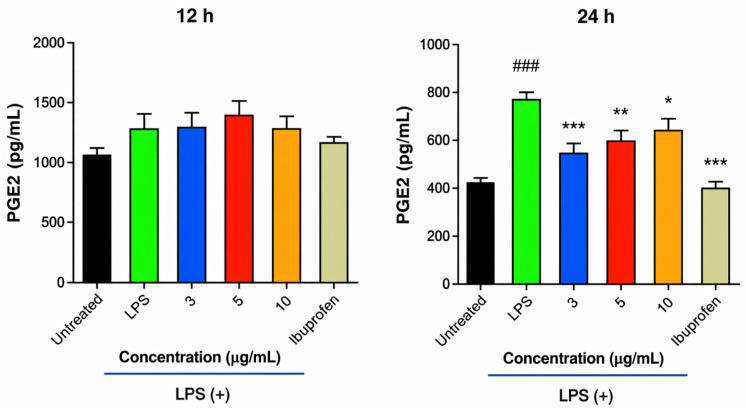
Effect of PS on the production of prostaglandins in LPS-stimulated macrophages after 12 and 24 h of treatment. PS did not have significant effects on prostaglandin production after 12 h of treatment but significantly decreased prostaglandins in all concentrations when compared to LPS-stimulated cells after 24 h of treatment. Ibuprofen also showed significant effects after 24 h of treatment. Data is presented as mean ± SE (*n* = 9). Data was analyzed using one-way analysis of variance (ANOVA), followed by Tukey’s multiple comparison post hoc test. Statistical significance was considered at *p* < 0.05. Statistical analyses: ^###^ *p* < 0.001 compared with the untreated sample. * *p* < 0.05, ** *p* < 0.01, and *** *p* < 0.001 were compared with LPS-stimulated cells. Experiments were conducted in triplicate and repeated in 3 independent studies.

The results obtained exhibited that 1 µg/mL of LPS from *E. coli* significantly stimulated production of prostaglandins compared to unstimulated macrophages (Figure 5). There was a decrease in prostaglandin levels after treatment with PS relative to LPS-stimulated cells in the 12 h treatment period, even though this was not significant (*p* > 0.05) (Figure 5). All the concentrations of PS showed a significant decrease (*p* < 0.05) in prostaglandin levels after 24 h of treatment compared to LPS-stimulated cells.

#### 2.4.3. Inhibition of Bovine Serum Albumin Denaturation

The anti-denaturing effects of PS on bovine serum albumin (BSA) protein were assayed in vitro as an anti-inflammation model. PS exhibited a concentration-dependent significant (*p* < 0.001) inhibition of BSA denaturation when compared to the untreated control after 20 min of treatment with an IC_50_ of 0.74 µg/mL. Importantly, PS at 10 µg/mL showed equivalent anti-inflammatory activity as the positive control, sodium diclofenac (66% versus 70% anti-inflammatory activity, *p* > 0.05) (Table 2).

### 2.5. Antimicrobial Activity

#### 2.5.1. Minimum Inhibition Concentration

The MICs of the boiled water and methanol extracts of PS are illustrated in Table 3. The boiled water extract of PS exhibited MIC values of 5000 µg/mL against *S. aureus* and 625 µg/mL for *S. epidermidis*. The methanolic extract had an MIC value of 5000 µg/mL against *S. aureus*, while for *S. epidermidis* the MIC was 2500 µg/mL. Two percent of DMSO, which served as a vehicle for the methanol extract, did not show any inhibitory effects on the tested bacterial strains. At tested concentrations, the MIC values of PS were not determined for *Klebsiella species*, *E. coli*, *P. aeruginosa*, and *B. subtitles.* The MIC values for doxycycline, a broad-spectrum antibiotic, ranged from 2 to 16 µg/mL for all bacterial strains tested. However, amoxicillin, a penicillin-based antibiotic, was only effective against *S. aureus*, *E. coli*, and *S. epidermidis*.

#### 2.5.2. Selectivity Index Versus Fibroblasts/Macrophages

Table 4 reports the selectivity index (SI) for the boiled water and methanolic extracts of PS against *S. epidermidis* and *S. aureus*, microorganisms that were susceptible to PS. Against *S. epidermidis*, the water extract showed greater antibacterial potency (MIC—250 µg/mL) than the methanol extract (MIC—625 µg/mL), yet both exhibited very low SI values (≤0.05 in fibroblasts; 0.01–0.05 in macrophages), indicating poor selectivity, i.e., measurable cytotoxicity at concentrations at or below those required for growth inhibition. Against *S. aureus*, both extracts had weak activity (MIC—5000 µg/mL) and again were non-selective (SI ≈ 0.05–0.06) against both fibroblasts and macrophages. No extract–pathogen combination achieved SI ≥ 1, indicating an unfavorable in vitro safety window under the present assay conditions. These interpretations follow standard antibacterial SI reporting (SI = LC_50_/MIC) and commonly used screening thresholds (SI ≥ 10 high; 3–9.9 moderate; 1–2.9 moderate; <1 poor).

#### 2.5.3. Zone of Inhibition

The results of this study demonstrated that PS exhibited inhibitory activity against selected bacterial strains associated with wound infections. Among the tested organisms, only *S. epidermidis* was susceptible to both the water and methanolic extracts of PS. The water extract of PS was notably more active against *S. epidermidis*, producing a zone of inhibition measuring 17 mm, which was greater than that observed with the methanolic extract at 10 mm (Table 5, Supplementary Figure S1). In contrast, *S. aureus*, *E. coli*, *K. pneumoniae* cK, *K. pneumoniae* HV, *P. aeruginosa*, and *B. subtilis* were resistant at the tested concentrations, with no zones of inhibition observed (Table 5, Supplementary Figure S1).

#### 2.5.4. Antibiofilm Activity

**Figure 6:**
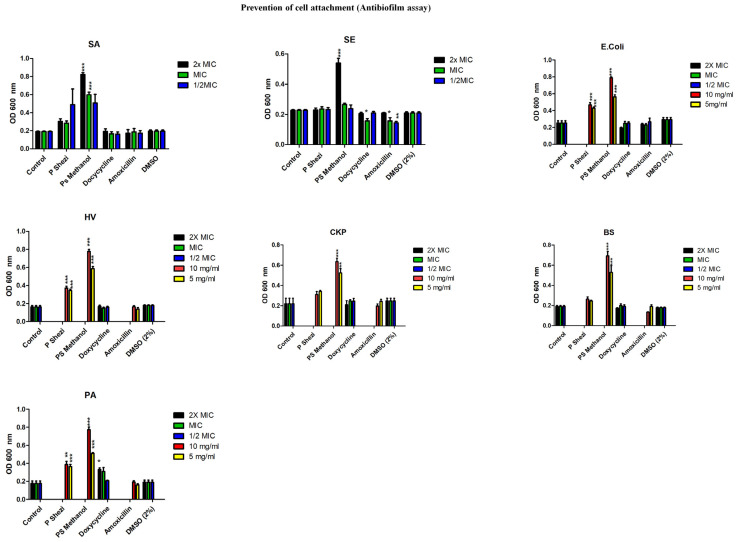
Effects of boiled water and methanol extracts of PS on biofilm formation by *S. aureus*, *S. epidermidis*, *E. coli*, *P. aeruginosa*, *B. subtilis*, *K. pneumoniae*, and cK and *K. pneumoniae* HV after 24 h of treatment. Both extracts of PS had no inhibitory effects on biofilm formation but were shown to promote biofilm formation in most of the microorganisms. Experiments were conducted in triplicate and repeated in three independent studies (*n* = 9). Statistical analysis was conducted using one-way ANOVA followed by Tukey’s post hoc test, where * *p* < 0.05, ** *p* < 0.01 and *** *p* < 0.001 compared to the untreated control.

**Figure 7:**
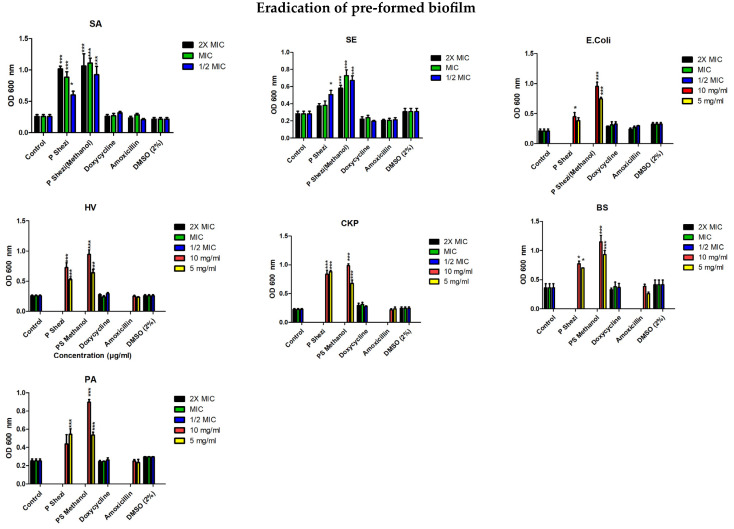
Effects of boiled water and methanol extracts of PS on the eradication of pre-formed biofilms by *S. aureus*, *S. epidermidis*, *E. coli*, *P. aeruginosa*, *B. subtilis*, *K. pneumoniae*, and cK and *K. pneumoniae* HV after 24 h of treatment. Both extracts of PS had no inhibitory effects on biofilm eradication but were shown to promote biofilm expansion in all the microorganisms. Experiments were conducted in triplicate and repeated in three independent studies (*n* = 9). Data were analyzed using one-way ANOVA followed by Tukey’s post hoc test, where * *p* < 0.05 and *** *p* < 0.001 compared to the untreated group.

The effect of PS on biofilm formation and development by *S. aureus*, *S. epidermidis*, *E. coli*, *P. aeruginosa*, *B. subtilis*, *K. pneumoniae*, and cK and *K. pneumoniae* HV is illustrated in Figure 6 and Figure 7. PS exerted a negative inhibitory effect on both biofilm formation and eradication in all tested microorganisms. The results demonstrated that PS promoted biofilm formation across all bacterial strains, as indicated by higher OD_600_ values than in untreated controls. In contrast, at least one of the reference antibiotics used in the study successfully inhibited biofilm formation, resulting in decreased OD_600_ values. Neither the water nor the methanolic extracts of PS were able to eradicate preformed biofilms in any of the tested microorganisms.

## 3. Discussion

This study aimed to evaluate the in vitro antioxidant, anti-inflammatory, and antimicrobial activities of Product Shezi (PS), a South African traditional medicine used for wound healing. Cytotoxicity evaluation revealed that the IC_50_ values of PS on the tested cell lines were below 50 µg/mL, suggesting that less extract is needed to reduce the viability of cell lines. This suggests that formulating herbal medicine with more than one plant species can enhance cytotoxicity effects and biological activity [30]. The observed cytotoxicity effects of PS on the tested cell lines could be ascribed to the presence of phytoconstituents such as saponins, flavonoids, alkaloids, and tannins, as demonstrated in the qualitative phytochemical screening of this traditional medicine [31].

Based on the cytotoxicity profile of PS, concentrations inducing 10% cytotoxicity (IC_10_) were used for efficacy evaluation using antioxidant, anti-inflammatory, and antimicrobial assays. In the antioxidant assays, PS exhibited strong antioxidant activity in the DPPH assay and through induction of cytoprotecting effects against stress induced by hydrogen peroxide. The observed effects could be due to the presence of phenolic and flavonoid compounds, as demonstrated by the qualitative phytochemical profiling of PS. Antioxidants are substances or compounds that have the potential to neutralize free radicals by donating a hydrogen or an electron, which is attributed to biological compounds such as phenolics and flavonoids [31]. Scavenging of hydrogen peroxide and reduction in the DPPH salt at higher concentrations demonstrate an ability to protect biological systems against reactive oxygen species (ROS). In wound healing, excessive production of ROS, such as peroxides, hinders the wound healing process [32]. For compounds to demonstrate stronger antioxidant activity, they must have stronger hydrogen donor groups [33]. Interestingly, it was observed that PS protected cells from H_2_O_2_ stress in both co-treatment and pre-treatment strategies. This suggests that PS is capable of preventing and neutralizing free radical formation to reduce oxidative damage during wound healing. Within the PS formulation, several constituent plants have been investigated for their antioxidant potential. Among the six species incorporated into PS, *B. latifolia* [34], *H. hemerocallidea* [28], *C. sativa* [35] and *D. altissima* [36] have been consistently documented to exhibit pronounced antioxidant activity. This bioactivity is primarily attributed to their relatively high levels of phenolic and flavonoid constituents, which are widely recognized for their ability to scavenge free radicals and mitigate oxidative stress [37]. Empirical studies further indicate that extracts from these species produce dose-dependent increases in antioxidant capacity, underscoring their pharmacological relevance. Collectively, the presence of these bioactive compounds suggests that the therapeutic efficacy of PS may, in part, be mediated by the antioxidant contributions of these plants, thereby supporting its traditional applications and providing a plausible scientific rationale for its use.

Anti-inflammatory agents derived from medicinal plants have been shown to aid the transition of wound healing phases from inflammation to remodeling [38]. In the anti-inflammatory assays, PS was not effective in reducing the production of nitric oxide in LPS-stimulated macrophages when compared to ibuprofen. Nitric oxide is a free radical that is synthesized by l-arginine and nitric oxide synthase (NOS) [39]. Inhibition of nitric oxide is an essential step to promote inhibition of the cyclooxygenase (COX) enzyme, thus reducing the levels of prostaglandins [40]. Nitric oxide and prostaglandins are mediator chemical substances that activate macrophage cells to coordinate an inflammatory response [41]. Therefore, the PGE_2_ ELISA assay served to confirm the downstream anti-inflammatory potential of PS. Prostaglandins are produced enzymatically from arachidonic acid by COX enzymes to control pain [42]. NO and PGE_2_ are regulated through related but distinct inflammatory pathways. NO production is mainly associated with inducible nitric oxide synthase (iNOS), whereas PGE_2_ production is primarily linked to the cyclooxygenase-2 (COX-2)/PGE_2_ pathway. It is therefore possible for a test substance to suppress PGE_2_ more effectively than NO, depending on its molecular targets, assay sensitivity, duration of stimulation, and the extent to which these pathways are induced under the experimental conditions [43,44,45,46]. In our study, the results suggest that Product Shezi may exert a stronger modulatory effect on the PGE_2_-associated inflammatory pathway than on NO production. PS significantly inhibited the production of PGE2, signifying anti-inflammatory effects. The inhibitory effect of PS against prostaglandin synthesis suggests that PS has the potential to reduce pain during the wound healing process. To further ascertain the anti-inflammatory potential of PS, a cell-free BSA denaturation assay was undertaken as a confirmatory assay. Denaturing of protein has been regarded as one of the outcomes of inflammation [47]. Proteins typically denature when they lose their secondary and tertiary structures due to external stress such as heat and organic solvents [48]. This study demonstrated the protein anti-denaturation effects of PS through a significant reduction in heat-induced BSA protein denaturation, suggesting strong anti-inflammatory activity at non-toxic concentrations. The observed effects can be linked to the ability of PS to inhibit the thermal unfolding and aggregation and neutralize free radicals. The anti-inflammatory potential of Product Shezi is supported mainly by the reduction in PGE_2_ production and BSA anti-denaturation activity, while the NO results did not show a significant inhibitory effect under the tested conditions.

There are interdependent relationships between antioxidants and inflammation [49]. There is a correlation between an increase in antioxidant activity and anti-inflammation [50]. The anti-denaturing effects can be due to the presence of phenolic compounds, as demonstrated in the qualitative phytochemical screening [51]. The results from this study suggest that PS could provide a balanced environment that will favor wound healing by reducing the inflammation phase while providing beneficial antioxidant effects. The observed anti-inflammatory effects can be attributed to the phytochemical constituents of the plants incorporated into the PS. *D. altissima*, *A. fastigiata*, *B. latifolia*, *H. hemerocallidea*, *H. aethiopicum*, and *C. sativa* have demonstrated notable anti-inflammatory properties, reinforcing their ethnopharmacological relevance in the management of chronic diseases associated with oxidative stress and inflammation [28,52,53,54,55,56]. For instance, *H. hemerocallidea* and *B. latifolia* extracts have been shown to attenuate inflammatory mediators, aligning with their traditional use in wound healing [54,57]. Similarly, *H. aethiopicum* and *C. sativa* are rich in bioactive compounds that suppress pro-inflammatory cytokines and modulate immune responses, while *D. altissima* and *A. fastigiata* contribute steroidal saponins and homoisoflavonoids with dual antioxidant and anti-inflammatory activity. Collectively, these findings highlight the therapeutic promise of these indigenous species, positioning them as valuable candidates for integrative approaches to inflammation-linked pathologies.

Wounds are associated with a high risk of microbial infection. *S. aureus*, *P. aeruginosa*, and *Streptococcus* spp. are bacteria that most commonly invade wounds within 3 days of injury and become the main source of infection [58]. These bacteria develop biofilms on wound sites that contribute to a delay in healing [59]. This biofilm contributes to the development of chronic wounds [60]. Bacterial infection and long-term inflammation have the potential to inhibit the wound healing process and subsequently delay the healing process [61]. Therefore, evaluation of the antimicrobial activities of the aqueous and methanolic extracts of PS was critical in further elucidating the contribution of this traditional medicine to wound healing. Previous studies have demonstrated that aqueous extracts generally exhibit limited antimicrobial activity, largely due to the fact that many bioactive phytochemicals responsible for antimicrobial effects are predominantly non-polar and therefore poorly extracted in aqueous solvent [62]. Therefore, the inclusion of methanolic extract was essential to enhance the antibacterial potential of PS, facilitating the extraction of bioactive constituents across solvents of varying polarity. Methanol extraction was performed specifically for antibacterial testing to explore whether compounds with stronger antibacterial activity could be recovered more effectively in an organic solvent than in water. Since the aqueous extract showed limited antibacterial activity, the methanolic extract was included to allow comparison of solvent-dependent extraction efficiency and its effect on antibacterial performance. Although aqueous (boiled water) extracts reflect traditional preparation methods in ethnomedicine, methanol is widely recognized as an effective solvent for extracting a broader spectrum of phytochemicals with antimicrobial activity [63]. These include moderately polar to less polar compounds such as phenolics, flavonoids, and terpenoids, which are frequently implicated in antimicrobial effects [64]. Consequently, methanolic extraction promotes the likelihood of recovering bioactive constituents that may not be efficiently extracted with water alone, thereby providing a more comprehensive evaluation of the formulation’s antibacterial properties. The present study demonstrated that both aqueous and methanolic extracts of PS exhibited selective antibacterial activity against the tested *S. epidermidis* and *S. aureus*. The observed inhibitory effect may be attributed to the presence of phytochemicals such as terpenoids and alkaloids. Previous studies have reported that terpenoids exert antimicrobial effects through mechanisms including the disruption of the bacterial cell wall, inhibition of quorum sensing, and interference with ATP synthesis [65,66]. Despite the observed antibacterial activity, both extracts exhibited extremely low selectivity index (SI) values (≤0.05 in fibroblasts and 0.01–0.05 in macrophages), indicative of poor therapeutic selectivity. These findings suggest that cytotoxic effects occur at concentrations equal to or lower than those required to inhibit bacterial growth [67]. An SI value below 10 is widely regarded as a threshold for limited therapeutic applicability, reflecting inadequate discrimination between antimicrobial efficacy and host cell toxicity [68]. Collectively, these results highlight a narrow therapeutic window and raise concerns regarding the potential safety and clinical relevance of the extracts for antimicrobial activity [69]. The medicinal plants comprising PS have been evaluated previously and shown to possess significant antimicrobial activity. Notably, bulb extracts of *H. hemerocallidea* exhibited pronounced antimicrobial effects, which are likely attributable to their phenolic and flavonoid content [70]. Extracts of *C. sativa*, including seed oil as well as methanolic extracts, have demonstrated antimicrobial activity against a variety of bacterial species [71]. *B. latifolia* has been shown to possess antimicrobial activity against selected microorganisms [72]. Different factors, such as the extraction method, types of solvents used, and the use of the medicinal plants comprising PS, may, therefore, be the cause of the reduced antimicrobial activities demonstrated in the current study.

In addition to the evaluation of the antimicrobial activities of PS against individual microorganisms, the current study demonstrated no antibiofilm activities of PS at the tested concentrations. On the contrary, both the boiled water and methanolic extracts of PS appeared to enhance biofilm attachment and development. This effect may be due to metabolites in PS that create favorable conditions for biofilm formation. It has been reported that some medicinal plants possess bioactive compounds that promote biofilm formation and production of efflux pumps [73]. An efflux pump is a mechanism utilized by bacteria to recognize and expel foreign, harmful substances that have entered through the cell wall [74]. In addition, the complex outer membrane structure of Gram-positive bacteria may prevent the extract from penetrating cells [75]. These could be some of the factors contributing to the resistance to the tested PS concentrations. The persistence of certain enzymes in the periplasmic space contributes to the resistance of biofilm to plant extracts [76]. Although PS inhibited *S. aureus* and *S. epidermidis* in their planktonic forms, it failed to inhibit biofilm formation at equivalent concentrations (see Figure 7) A similar effect was observed with other medicinal plant extracts in a previous study [77]. This study demonstrated that extracts from *Betula pendula* reduced OD600 values in the planktonic fraction without decreasing total biofilm biomass, suggesting that they enhanced biofilm formation [78]. The results suggest that PS was not sufficiently bactericidal to eliminate bacterial cells and may instead have induced stress responses that promote biofilm development. It has been proposed that plants with high phenolic content may enhance biofilm development [79]. Based on these findings, PS may be more suitable for promoting wound healing when combined with antibiotics to enhance infection control and improve therapeutic outcomes. Some PS component plants, such as *C. sativa*, have been reported to exhibit strong antibiofilm activity against *S. aureus* at minimum inhibitory concentration levels [80]. However, complete inhibition of biofilm formation was not observed. Similarly, the dichloromethane extract of *H. hemerocallidea* has been shown to inhibit initial cell attachment of *S. aureus* and *Serratia marcescens* [81]. Collectively, these findings suggest that while individual plant constituents may contribute to antibiofilm effects, their activity is often partial, supporting the potential value of PS as an adjunct rather than a standalone antimicrobial therapy.

## 4. Materials and Methods

### 4.1. Preparation of Traditional Medicine and Plant Materials

#### 4.1.1. Product Shezi Water Extract

The traditional medicine, Product Shezi (PS), was prepared according to the instructions of the traditional healer. The traditional medicine product corresponds to a Type C extract, defined as a polyherbal formulation derived from lesser-studied botanical species that are not listed in pharmacopeias or widely commercialized. PS is a polyherbal ATM formulated from six medicinal plants, namely *Drimia altissima* (L.f.). Ker Gawl., *Albuca fastigiata* Dryand, *Bulbine latifolia* (L.f) Spreng, *Hypoxis hemerocallidea* Fisch., C.A. & Ave-Lall, *Hypericum aethiopicum* Thumb, and *Cannabis sativa* L. Specimens of these medicinal plants were collected from Richmond (29°54′ 14.79″ S, 30°07′ 44.97″ E) in the uMgungundlovu District Municipality, KwaZulu-Natal by the traditional healer, Mr Shezi, who disclosed the traditional uses of the plants. A permit for the use of medicinal cannabis was obtained from the South African Health Products Regulatory Authority (SAHPRA) (Supplementary Figure S2). The medicinal plants were verified and classified by Dr Syd Ramdhani, a curator in the Herbarium of the School of Life Sciences at the University of KwaZulu-Natal, and specimens were deposited at the Bews Herbarium in Pietermaritzburg, as shown in Table 6.

To prepare PS according to the instructions of the traditional healer, the plant material from the six individual medicinal plants was sun-dried and ground into powder using a mechanical grinder. The powders from the five medicinal plants were combined at a 1:1 ratio to make up 50% of the final ATM product, while *Cannabis sativa* was added to make up the remaining 50%. The combined plant material was boiled for 2 h and cooled to room temperature. The aqueous extract was initially filtered through Whatman No. 1 filter paper, then lyophilized using a VirTis SP Scientific freeze dryer (SP Scientific, Warminster, PA, USA) to obtain a dry powder. A stock solution was prepared by dissolving 50 mg of the lyophilized extract in 5 mL of phosphate-buffered saline (PBS), followed by sterile filtration through a 0.22 µm Corning filtration system (Corning Incorporated, Corning, NY, USA). Serial dilutions were subsequently prepared in Dulbecco’s Modified Eagle Medium (DMEM) supplemented with fetal bovine serum (FBS) and penicillin–streptomycin for use in downstream assays.

#### 4.1.2. Product Shezi Methanolic Extract

For the antimicrobial assays, a methanolic extract of PS was prepared for comparative purposes. The methanolic extract was included specifically to assess whether extraction with an organic solvent could improve the recovery of compounds associated with antimicrobial activity, particularly because the aqueous extract showed limited antibacterial effects. The dried plant material received from the traditional healer was mixed at similar ratios as Section 2.1 above, with *C. sativa* making up 50% of the final weight. The mixture of the powdered plant material (20 g) was then extracted with 200 mL of 100% HPLC grade methanol by maceration at room temperature for 48 h under continuous agitation. Following extraction, the mixture was filtered through Whatman No. 1 filter paper to remove particulate matter. The filtrate was then evaporated to dryness at room temperature under a fume hood to obtain the crude methanolic extract. For in vitro assays, the dried extract was reconstituted in 2% dimethyl sulfoxide (DMSO) to prepare a 10 mg/mL stock solution.

### 4.2. Qualitative Phytochemistry Analysis

Qualitative phytochemical screening of both the boiled and methanolic extracts of PS was performed using standard qualitative assays to identify major classes of secondary metabolites. Alkaloids were detected using Mayer’s, Dragendorff’s, and Hager’s reagents; flavonoids were identified using the Shinoda test; saponins were evaluated using the froth test; phenolic compounds were assessed using Liebermann’s test; and terpenoids were detected using the Salkowski test. Observations were based on characteristic color changes or precipitate formation.

i. Test for Flavonoids

An aliquot of 500 µL of each PS extract was added to separate 15 mL tubes, followed by the addition of 500 µL of ammonia solution. Finally, 100 µL of sulfuric acid (H_2_SO_4_) was added to the reaction mixture. The appearance of a yellow color indicated the presence of flavonoids [81].

ii. Test for Saponins

A 500 µL aliquot of each extract was added to separate 15 mL tubes, followed by 250 µL of boiling water. The mixture was vortexed and allowed to rest for 10 min at room temperature. The appearance of a persistent froth indicated the presence of saponins [82].

iii. Test for Tannins and Phenols

A volume of 500 µL of each extract was added to separate 15 mL tubes, followed by 100 µL of 2% ferric chloride solution. The appearance of a blue–black or greenish-black precipitate indicated the presence of tannins and phenols [83].

iv. Test for Glycosides

A volume of 500 µL of each extract was added to separate 15 mL tubes, followed by 200 µL of chloroform and 200 µL of acetic acid. The reaction mixture was cooled on ice for 5 min. After cooling, 150 µL of sulfuric acid (H_2_SO_4_) was added. The development of a green coloration indicated the presence of glycosides [81,84].

v. Test for Steroids

A volume of 500 µL of each extract was added into separate 15 mL tubes, followed by 200 µL of chloroform and 150 µL of H_2_SO_4_. The formation of a red coloration in the lower chloroform layer indicated the presence of steroids [81,83].

vi. Test for Alkaloids

A volume of each extract was added to separate 15 mL tubes, followed by 500 µL of hydrochloric acid (HCl). The reaction mixture was then placed in a boiling water bath for 5 min. After cooling, four drops of Dragendorff’s reagent were added. The formation of turbidity or a precipitate indicated the presence of alkaloids [81].

### 4.3. Cell Culture

Rat fibroblast (CFRK-RM) and Murine RAW-264.7 macrophage cells were purchased from Cellonex (Gauteng, South Africa) and maintained in Dulbecco’s Modified Eagle’s Medium (DMEM), supplemented with 10% FBS and 1% Penicillin Streptomycin in a humidified incubator at 37 °C and 5% CO_2_. These cells were subcultured twice a week and seeded into 96- or 48-well plates for experiments when at 80–95% confluence.

### 4.4. Cytotoxicity Assay (ATP Assay)

An adenosine triphosphate (ATP)-based luminescence assay was used to evaluate the cytotoxic effects of PS. This method quantifies intracellular ATP levels as an indicator of metabolically active and viable cells. The luminescent signal is directly proportional to the number of viable cells present. For our experiments, fibroblast and macrophage cells were seeded into 48-well plates at a density of 1.0 × 10^5^ cells per well and allowed to attach overnight under standard cell culture conditions. Subsequently, the cell lines at ±80% confluency were treated with PS at concentrations ranging from 0 to 250 µg/mL. Untreated cells maintained in complete DMEM served as negative controls. Following treatment, the cells were incubated for 24 h at 37 °C and 5% CO_2_. At the end of a 24 h incubation period, the effects of PS on cell viability were quantified with the CellTiter-Glo^®^ assay kit (Promega, Madison, WI, USA), following the manufacturer’s instructions. Briefly, at the end of the incubation period, each cell line was harvested via trypsinization, and 100 µL from each well was transferred into an opaque white 96-well microplate. An equal volume of CellTiter-Glo^®^ Reagent was added to the well, covered by foil, and mixed for 2 min to induce cell lysis. The plate was incubated at room temperature away from light for 10 min, and the Glow-Max luminometer (Promega, Madison, WI, USA) was used to read the plate. Cell viability was expressed as a percentage relative to untreated control cells. IC_10_ values were calculated from dose–response curves for each cell line and used for further experiments.

### 4.5. Antioxidant Activity

#### 4.5.1. DPPH Radical Scavenging Assay

The free radical scavenging activities of PS were measured using the 2,2-diphenyl-1 picrylhydrazyl (DPPH) radical scavenging assay. Scavenging activity is assessed by observation of DPPH purple color reduction to yellow after addition of the extract in a dose-dependent manner [82]. A volume of 150 µL PS at various concentrations (1–10 µg/mL) was mixed with 50 µL DPPH solution in a 96-well plate. Ascorbic acid was used as a reference antioxidant. The mixture was incubated in the dark at room temperature (25 °C) for 30 min to prevent the light-induced degradation of DPPH. Subsequently, the absorbance was measured at 517 nm using a GloMax^®^ Explorer Multimode Microplate Reader (Promega, Madison, WI, USA). The assay was carried out in triplicate wells and repeated 3 times. The percentage inhibition of DPPH free radical was calculated using the following equation:

Percentage Inhibition (%) = [(A_517_ control − A_517_ sample)/A_517_ control] × 100

IC_50_ values were determined using nonlinear regression in GraphPad Prism 8.0. Data are presented as mean ± SD.

#### 4.5.2. Hydrogen Peroxide Assay

The cytoprotective effects of the PS against hydrogen peroxide (H_2_O_2_) induced oxidative damage were evaluated as described by Ustuner et al. [9]. Fibroblast cells were seeded into 96-well plates at a density of 1.0 × 10^4^ cells/well and incubated for 24 h at 37 °C and 5% CO_2_. The cells were initially treated with increasing concentrations of H_2_O_2_ (0–0.04 µM) and incubated for 3 h. At the end of the incubation period, cell viability was determined using the CellTiter-Glo^®^ assay kit (Promega, Madison, WI, USA) as described in Section 2.4 above. Percent cell viability was calculated, and the dose that induced 50% cell death was used for the induction of oxidative stress. Two treatment strategies were evaluated to determine the cytoprotective effects of the extracts against hydrogen peroxide-induced oxidative stress. In the pre-treatment regimen, cells were first exposed to non-cytotoxic concentrations of the extract for 24 h, followed by the addition of 10^−4^ M H_2_O_2_ for 3 h. In the co-treatment regimen, cells were simultaneously treated with the extract concentrations and 10^−4^ M H_2_O_2_ for 3 h. Ascorbic acid (10 µg/mL) was used as a positive control. At the end of the incubation period, the CellTiter-Glo^®^ assay kit was used to determine the protective effects of the extract against H_2_O_2_-induced oxidative stress in fibroblast cells.

### 4.6. Anti-Inflammation

#### 4.6.1. Inhibition of BSA Denaturing

The method described by Zhang et al. [29] was used to analyze the in vitro anti-inflammatory activity of PS. Briefly, 1% *w*/*v* aqueous solution of bovine serum albumin (BSA) was prepared by dissolving 0.5 g of BSA in 50 mL of deionized water. For experiments, a reaction mixture consisting of 0.45 mL of 1% BSA solution and various non-cytotoxic concentrations of PS in separate test tubes was prepared. The tubes were incubated for 20 min at 37 °C and then heated for 5 min at 53 °C. After the samples were cooled, 2.5 mL of PBS was added to each tube. The absorbance was then measured at 660 nm. Deionized water was used as a negative control, and diclofenac sodium was used as a standard anti-inflammatory drug. The protein denaturation inhibition percentage was determined by the following formula:

Percentage Inhibition (%) = [(_A660_ control − A_660_ sample)/A_660_ control] × 100

IC_50_ values were determined using nonlinear regression in GraphPad Prism 8.0. Data are presented as mean ± SD.

#### 4.6.2. Nitric Oxide Production Inhibition Assay

The Griess reagent system was used to further confirm the anti-inflammatory effects of PS. Briefly, 200 μL of actively growing macrophage suspension was seeded into each of the 96-well plates overnight to form a monolayer. After incubation, the medium was removed from the cells and replaced with DMEM containing 1 µg/mL of LPS. The cells were incubated for 3 h to allow for the induction of inflammation. After incubation, cells were treated with various concentrations (1–10 µg/mL) of PS without removing the LPS stimuli and incubated for 12 and 24 h. A nonsteroidal anti-inflammatory drug, ibuprofen (10 µg/mL), was used as the standard drug. After each incubation period, the Griess reagent system was used to quantify the amount of nitric oxide in culture supernatant following the manufacturer’s instructions. Briefly, 50 µL of supernatant was transferred into the 96-well plate, then 50 µL of 1% sulfanilamide was added. The plate was covered with foil and incubated at room temperature for 10 min. After the addition of 50 µL of 0.1% naphthyl ethylenediamine dihydrochloride, a pink color immediately developed. Optical density was measured spectrophotometrically at 570 nm.

#### 4.6.3. The Effects of ATM on PGE2 Production

The inhibitory effects of PS on prostaglandin E2 (PGE_2_) production were quantified using an Invitrogen™ Human Prostaglandin E2 ELISA Kit (ThermoFisher Scientific, Fairland, Johannesburg, South Africa). The Invitrogen™ Human Prostaglandin E_2_ ELISA Kit is a competitive enzyme-linked immunosorbent assay designed to measure and quantify the concentration of prostaglandin E_2_ (PGE_2_) in human biological samples. Briefly, RAW 264.7 macrophage cells were cultured in T75 flasks until 80% confluence. After harvesting cells via trypsinization, the cells were seeded at a concentration of 1.0 × 10^6^ cells/well in 24-well plates and incubated for 24 h at 37 °C and 5% CO_2_. After the incubation period, the medium of each well was removed, and the cells were stimulated with 1 µg/mL of LPS for 3 h. Following stimulation, cells were treated with various concentrations (1–10 µg/mL) of PS and ibuprofen (10 µg/mL) without removing the LPS stimuli. The cells were incubated for 12 and 24 h. After each incubation period, culture supernatants were collected and centrifuged at 3000 rpm for 10 min to remove debris. PGE_2_ concentrations were determined according to the manufacturer’s instructions. Next, 100 µL of each sample and the prostaglandin E2 (PGE_2_) standard were added to the appropriate wells of the ELISA plate. Subsequently, 50 µL of prostaglandin E2 alkaline phosphatase tracer was added, followed by 50 µL of prostaglandin E2 antibody. The plate was covered and incubated for 2 h on an orbital shaker. After washing the wells five times, 200 µL of pNPP substrate solution was added to each well. The plate was covered and incubated in the dark on an orbital shaker for 1 h, and absorbance was measured at 405 nm using a GloMax^®^ Explorer Multimode Microplate Reader.

### 4.7. Antimicrobial Activity

#### 4.7.1. Bacterial Culture

Gram-positive and Gram-negative bacterial strains were obtained from the Discipline of Medical Microbiology, University of KwaZulu-Natal. The standard bacterial strains included *Bacillus subtilis* (BS), *Staphylococcus aureus* (SA), *Escherichia coli* (*E. coli*), *Pseudomonas aeruginosa* (PA), *Classical Klebsiella pneumonia* (cKp), *Hypervirulent Klebsiella pneumonia* (hvKp), and *Staphylococcus epidermidis* (SE). These strains were selected due to their known involvement in wound infections. As bacterial wound infections are considerably more prevalent than fungal wound infections, this study focused primarily on bacterial pathogens [19,57,85]. Overnight cultures were prepared by inoculating 1 mL of each bacterial strain into 10 mL of sterile nutrient broth medium. The cultures were incubated aerobically at 37 °C. Bacterial wound infections are considerably more prevalent than fungal wound infections; therefore, this study focused primarily on bacterial pathogens.

#### 4.7.2. Determination of Minimal Inhibition Concentration

The method described by Ramli et al. [86] was used to determine the minimal inhibition concentration (MIC). The assay was conducted in a 96-well plate using a twofold standard broth micro-concentration dilution resazurin-based method with an inoculum of 0.5 McFarland standards. Briefly, bacterial strains known to infect wounds were first cultured in broth medium overnight at 37 °C. Following the incubation period, bacteria were adjusted to a final 0.2 OD of McFarland standards. A two-fold dilution of PS and reference drugs (doxycycline and amoxicillin) with a concentration range from 5000 µg/mL to 4.88 µg/mL was prepared, serially diluted in the respective medium. Column 2 of the microtiter plate contained the highest concentration of PS (5000 µg/mL), while column 11 contained the lowest concentration of the diluted product (4.88 µg/mL). Dilution for the reference drug started from the highest concentration of 1 mg/mL. Column 1 served as a negative control (only medium, no inoculum, and no antimicrobial agent), while column 12 served as a positive control for all samples (only medium and inoculum or antimicrobial agent-free well) for 24 h. The plates were then incubated aerobically at 37 °C for 24 h. At the end of the incubation period, 30 µL of the resazurin dye was added to the 96-well plate to detect the growth of the bacterium by color change. A column with living bacteria oxidized the blue color of resazurin dye to pink. The column with no metabolically active cells remained blue. The MIC was defined as the lowest concentration of antimicrobial agent that was able to inhibit visible growth. All experiments were carried out in triplicate and repeated on three independent occasions.

#### 4.7.3. Anti-Bacterial Activity

The antibacterial activity of PS was assessed using the agar well diffusion method. Gram-positive and Gram-negative bacteria affecting wounds were cultured in Mueller–Hilton broth (HiMedia) medium overnight at 37 °C. The Mueller–Hilton agar medium (HiMedia) was prepared and poured aseptically into the sterile Petri plates. The respective bacterial strains were adjusted to match the final optical density (OD) of 0.5 in HiMedia. Furthermore, 100 µL of bacterial pathogens (OD 0.5) was swabbed into the agar plate wells using a sterile L-shape cell spreader. Next, agar plates with inoculum were allowed to dry out for 10–15 min in a 37 °C incubator. Then, the equidistant wells with a diameter of 8.0 mm were bored with a cork, and the wells were filled with 100 µL of the different concentrations of PS, and a standard antibiotic was used for wound healing. Different concentrations of the PS, as well as standard antibiotics (doxycycline and amoxicillin), were added into the respective wells, and the plates were incubated at 37 °C for 24 h. After incubation, the zones of inhibition were measured in millimeters (mm) using a ruler.

#### 4.7.4. Antibiofilm Activity

The effects of the PS and standard antibiotics on the formation and development of biofilms formed by the tested bacteria on wound surfaces were evaluated using the crystal violet assay. The experiments were divided into two approaches: prevention of biofilm formation and eradication of preformed biofilms. Two separate 24-well plates were prepared for each bacterial strain. The first plate was designated for evaluating the effect of PS on biofilm formation, while the second plate was used to assess the eradication of established biofilms. For the prevention strategy, 1 mL of overnight-grown bacterial cultures were inoculated into 10 mL of Soutanes’ medium and incubated under standard conditions. Biofilm formation was initiated by pipetting 1 mL of the respective culture (OD_600_ = 0.1 or 1.0 × 10^6^ CFU/mL) into sterile, flat-bottomed 24-well plates. Subsequently, 1 mL of PS (at sub-MIC concentration; for extracts without MIC, concentrations of 10 mg/mL and 5 mg/mL were used) was added to the wells containing the inoculum. Doxycycline and amoxicillin (at sub-MIC concentrations, for antibiotics without MIC, concentrations of 10 mg/mL and 5 mg/mL were used) were also added. The plates were incubated for 24 h at 37 °C without shaking.

The same principle was applied for the eradication strategy, except that biofilms were first allowed to form over 24 h before being treated with PS or standard antibiotics. After incubation, modified crystal violet staining was performed to assess the biofilm biomass.

### 4.8. Crystal Violet Staining

The 24-well plates were carefully emptied and washed three times with PBS to remove planktonic bacteria. The adhered cells were then stained with 1 mL of 0.1% crystal violet solution for 15 min at room temperature, while covered with microtiter plate lids. After staining, the plates were washed three times with PBS to remove excess dye. Biofilm biomass was evaluated semi-quantitatively by resolubilizing the crystal violet bound to the adherent cells using 1 mL of 30% acetic acid. After gentle shaking, the absorbance was measured at 600 nm using a microplate reader. The mean absorbance at 600 nm (OD_600_) was calculated, and three independent experiments were performed for each bacterial strain.

### 4.9. Statistical Analyses

All experiments were performed in technical triplicate and independently repeated at least three times (*n* = 3 biological replicates unless otherwise stated). Data are reported as mean ± standard deviation (SD). Statistical analyses were conducted in GraphPad Prism 8.0 (GraphPad Software, La Jolla, CA, USA). Data normality was assessed using the Shapiro–Wilk test. For comparisons among multiple groups with normally distributed data, one-way analysis of variance (ANOVA) followed by Tukey’s multiple comparisons test was applied. Two-sided *p*-values < 0.05 were considered statistically significant. Dose–response relationships and IC_50_/IC_10_ values were determined by nonlinear regression using a variable-slope, four-parameter logistic model [log(inhibitor) vs. normalized response] in GraphPad Prism 8.0. IC_50_ values for DPPH and BSA were estimated from the monotonic portion of each concentration–response curve.

## 5. Conclusions

Product Shezi has the potential to neutralize ROS production during wound healing, thereby promoting complete wound healing within a limited period. This ATM product was also shown to contain bioactive compounds that regulate inflammation by inhibiting protein denaturation and prostaglandin production. Product Shezi showed no significant antimicrobial activity at the tested concentrations; however, different extraction methods may yield better results. This study scientifically supported the use of PS as a traditional medicine for wound healing. Further studies should focus on direct wound healing activities in vitro and confirmatory studies using in silico and animal models.

## 6. Study Limitations

The present study has several limitations. First, the evaluation of the antioxidant, anti-inflammatory, and antimicrobial activities of PS was conducted exclusively in vitro, which may not fully reflect their biological effects in vivo. Therefore, future studies should include in vivo experiments to better assess the wound healing potential of PS. Secondly, only two extraction solvents were employed in the antimicrobial activity assays; thus, future work should explore a wider range of solvents with varying polarities, such as ethanol, ethyl acetate, acetone, chloroform, and hexane, to improve the recovery of bioactive constituents. Thirdly, phytochemical analysis in this study was limited to qualitative screening, which restricts the ability to correlate specific compounds with observed biological activities. Accordingly, future studies should incorporate advanced chromatographic techniques, such as thin-layer chromatography (TLC), high-performance liquid chromatography-mass spectrometry (HPLC–MS), together with quantitative phytochemical analyses, to enable the precise identification and quantification of bioactive compounds and a better understanding of their mechanisms of action. A limitation of this study is that the anti-inflammatory activity was assessed only at the selected time point(s), which may not have fully captured the delayed or time-dependent effects of the extract and positive controls; therefore, future studies should consider extended evaluations at 48 h. A limitation of this study is that only bacterial pathogens associated with wound infections were evaluated. Future studies should include antifungal screening to provide a more comprehensive assessment of the extract’s antimicrobial potential.

## Figures and Tables

**Table 1:** Phytochemical parameters of Product Shezi.

Compounds	Present/Absent
Flavonoids	+
Saponins	+
Tannins	+
Glycoside	+
Terpenoid	+
Carbohydrate	+
Steroids	+
Alkaloids	+

(+) = Present.

**Table 2:** Effect of PS on the inhibition of BSA protein denaturation. PS inhibited BSA denaturation in a significant dose-dependent manner when compared to the untreated control, with an IC_50_ of 0.74 µg/mL. Data was analyzed using one-way analysis of variance (ANOVA), followed by Tukey’s multiple comparison post hoc test. Statistical analyses: *** *p* < 0.001 compared with the untreated sample.

	Concentration µg/mL	Percentage Denaturation Inhibition
**Untreated**	0	0
**Product Shezi**	3	51 ***
	7	61 ***
	10	66 ***
**Diclofenac Na^+^**	50	70 ***

**Table 3:** The MIC values of PS extracts against standard bacterial strains causing wound infections. The MIC values for the boiled water extract were 5000 µg/mL against *S. aureus* and 625 µg/mL against *S. epidermidis*. The methanolic extract had an MIC of 5000 µg/mL against *S. aureus*, while against *S. epidermidis*, the MIC was 2500 µg/mL. Doxycycline was effective against all bacterial strains, whereas amoxicillin was specific for *S. aureus*, *E. coli*, and *S. epidermidis*. Experiments were conducted in triplicate and repeated in three independent studies.

	Minimum Inhibition Concentration (µg/mL)						
	*S. aureus*	*E. coli*	*S. epidermidis*	*Klebsiella cKp*	*Klebsiella hvKp*	*P. aeruginosa*	*B. subtills*
Doxycycline	2	2	2	8	8	16	16
Amoxicillin	4	4	2	-	-	-	-
Product Shezi water extract	5000	-	625	-	-	-	-
Product Shezi methanol extract	5000	-	2500	-	-	-	-
DMSO (2%)	-	-	-	-	-	-	-

(-) represents not determined.

**Table 4:** Selectivity index (SI) of PS against *S. epidermidis* and *S. aureus*.

Product Shezi	Pathogen	MIC (µg/mL)	SI (Fibroblast)	IC_50_ (Fibroblast, µg/mL)	SI (Macrophage)	IC_50_ (Macrophage, µg/mL)	Interpretation
Water	*S. epidermidis*	625	0.04	23.68	0.05	32.47	Poor selectivity
Water	*S. aureus*	5000	0.005	23.68	0.006	32.47	Poor selectivity
Methanol	*S. epidermidis*	2500	0.01	23.68	0.01	32.47	Poor selectivity
Methanol	*S. aureus*	5000	0.005	23.68	0.006	32.47	Poor selectivity

**Table 5:** Antimicrobial activity of PS against microbial organisms causing wound infection. Among the tested organisms, only *S. epidermidis* was susceptible to both the water and methanolic extracts of PS. Doxycycline induced zones of inhibition against all bacterial strains, while amoxicillin was specific for *S. aureus*, *E. coli*, and *S. epidermidis*. Experiments were conducted in triplicate and repeated in three independent studies. Data were analyzed using one-way ANOVA followed by Tukey’s post hoc test. *p* < 0.01 and *p* < 0.001 compared with the untreated group. ** *p* < 0.01 and *** *p* < 0.001 compared to 2% DMSO.

Diameter of Zone of Inhibition (mm)													
Microbes	Doxycycline			Amoxicillin			Product Shezi (Water)			Product Shezi (Methanol)			DMSO
	1000 µg/mL	100 µg/mL	10 µg/mL	1000 µg/mL	100 µg/mL	10 µg/mL	5000 µg/mL	500 µg/mL	50 µg/mL	5000 µg/mL	500 µg/mL	50 µg/mL	2%
*S. aureus*	30 ± 2.7 ***	16 ± 6.3 ***	12 ± 2.1 ***	20 ± 1.5 ***	10 ± 1.7 ***	10 ± 1.5 ***	NI	NI	NI	NI	NI	NI	NI
*E. coli*	31 ± 0.9 ***	21 ± 0.9 ***	12 ± 2.5 ***	33 ± 1.5 ***	25 ± 1.5 ***	13 ± 1.6 ***	NI	NI	NI	NI	NI	NI	NI
*S. epidermidis*	32 ± 2.2 ***	23 ± 3.6 ***	18 ± 2.4 ***	35 ± 1.8 ***	28 ± 0.9 ***	20 ± 0.9 ***	17 ± 1.2 **	NI	NI	10 ± 1.2 ***	NI	NI	NI
*Klebsiella cKp*	16 ± 0.9 ***	8 ± 0.9 ***	NI	NI	NI	NI	NI	NI	NI	NI	NI	NI	NI
*Klebsiella hvKp*	25 ± 1.6 ***	13 ± 4.3 ***	NI	NI	NI	NI	NI	NI	NI	NI	NI	NI	NI
*P. aeruginosa*	22 ± 4.8 ***	NI	NI	NI	NI	NI	NI	NI	NI	NI	NI	NI	NI
*B. subtitles*	20 ± 2.6 ***	12 ± 3.3 ***	NI	NI	NI	NI	NI	NI	NI	NI	NI	NI	NI

NI = No Inhibition (0 mm).

**Table 6:** Medicinal plants used in the formulation of Product Shezi.

Plant Name	Voucher Number
*Drimia altissima* (L.f.) Ker Gawl.	UDW23016
*Albuca fastigiata* Dryand	UDW23017
*Bulbine latifolia* (L.f) Spreng	UDW23018
*Hypoxis hemerocallidea* Fisch., C.A. & Ave-Lall	UDW23019
*Hypericum aethiopicum* Thunb	UDW23020
*Cannabis sativa* L.	UDW23021

## Data Availability

Data related to this article is contained in the article.

## Supplementary Materials

The following supporting information can be downloaded at https://www.mdpi.com/article/10.3390/ijms27156815/s1.
