# Supplementary material for: Evaluation of the Antioxidant, Anti-Inflammatory, and Antimicrobial Activity of a Cannabis sativa-Infused African Product Used for Wound Healing
Source: Int J Mol Sci. 2026 Jul 29;27(15):6815. doi: 10.3390/ijms27156815 (PMC13467544; doi:10.3390/ijms27156815)
Supplement: Supplementary file 1 [file ijms-27-06815-s001.zip › Supplementary Figures.pdf]

Supplementary Figures

	5000μg/mL or 1000μg/mL	500μg/mL or 100μg/mL	50μg/mL or 100μg/mL
E. coli	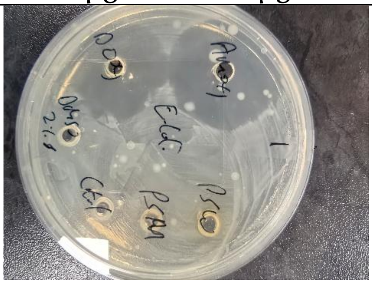 *A1*	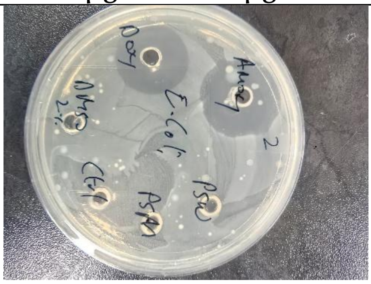 *A2*	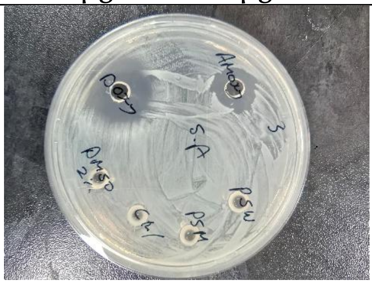 *A3*
S. aureus	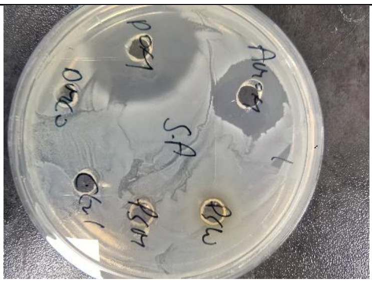 *B1*	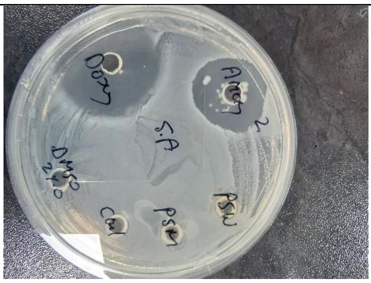 *B2*	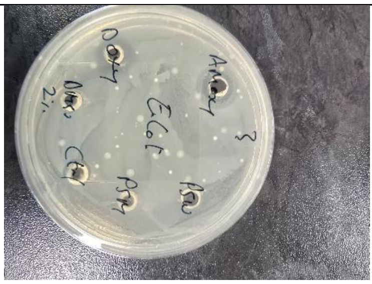 *B3*
S. epidermidis	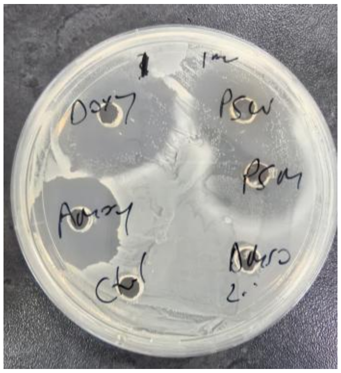 *C1*	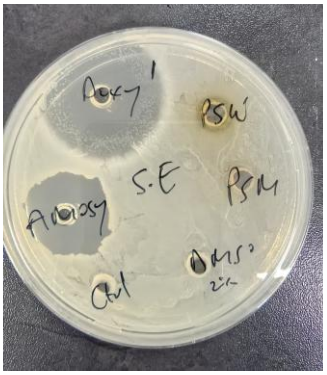 *C2*	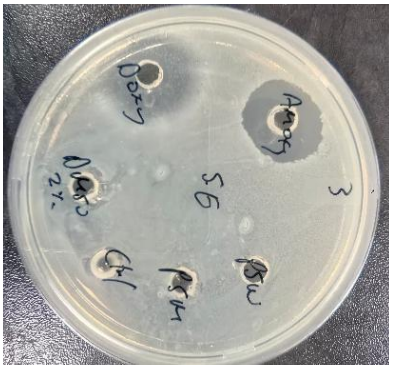 *C3*
Klebsiella hvKp	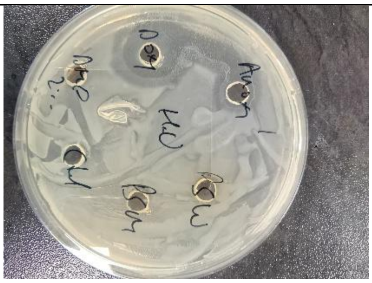 *D1*	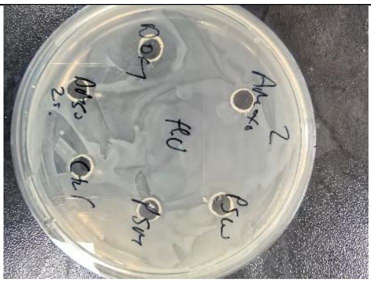 *D2*	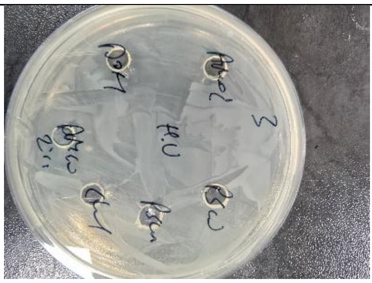 *D3*

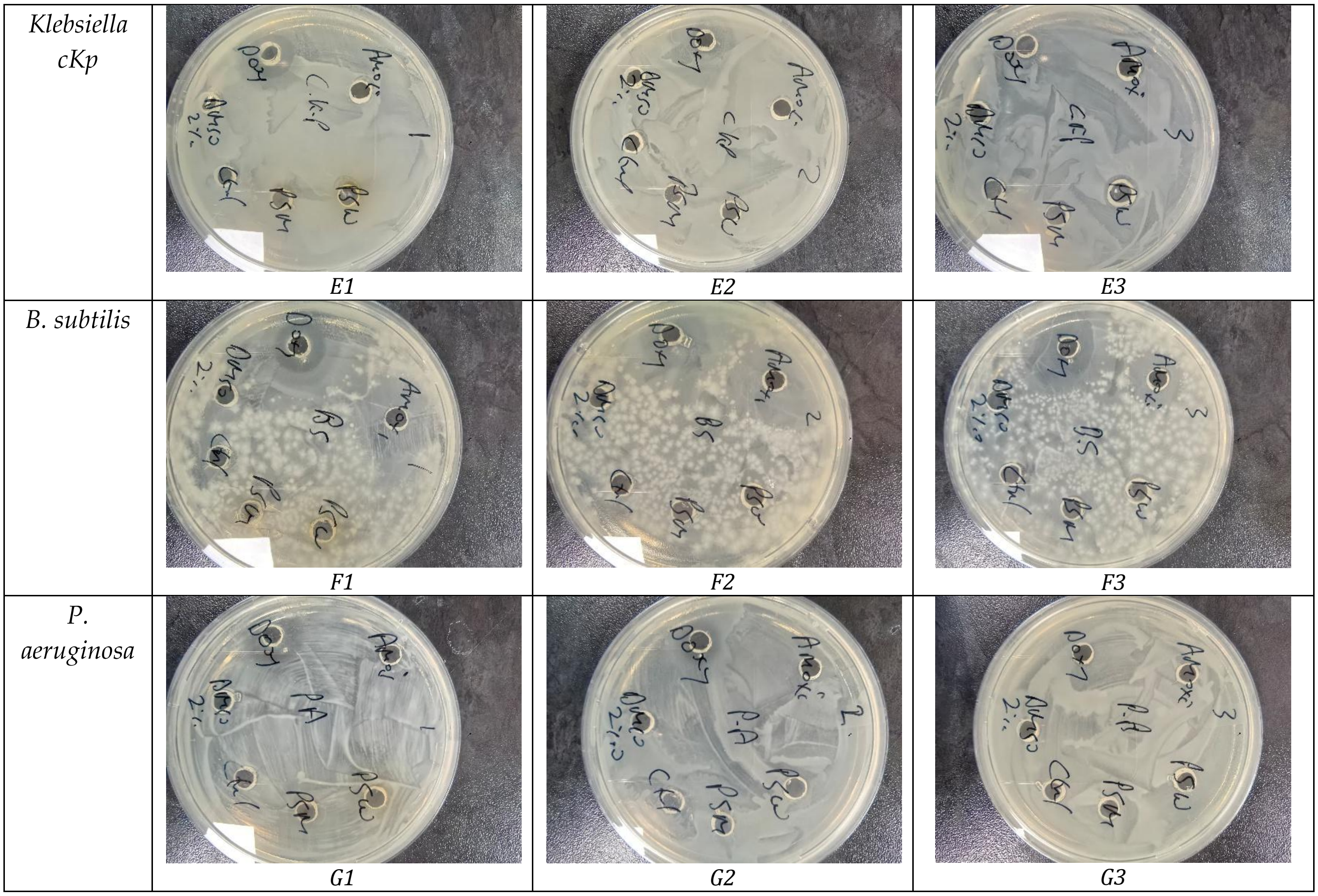

Supplementary Figure S1. Agar diffusion plates showing zones of inhibition at the indicated concentrations. For the extracts, a 10-fold serial dilution was used, ranging from 5000 to 50 μg/mL. For the standard antibiotics, concentrations ranged from 1000 to 10 μg/mL. *S. epedermidis* was the only microorganism susceptible to PS water and methanol extracts. Plate labels: PSW = Product Shezi (aqueous extract); PSM = Product Shezi (methanolic extract); Doxy = Doxycycline; Amoxy = Amoxicillin; Crt = Control; DMSO = Dimethyl sulfoxide.

health
Department:
Health
REPUBLIC OF SOUTH AFRICA

DEPARTMENT OF HEALTH
Private Bag X828
PRETORIA, 0001
Republic of South Africa

UMNYANGO WEZEMPILO
LEFAPHA LA MAPHELO

PERMIT TO ACQUIRE, POSSESS AND USE SPECIFIED SCHEDULE 5, SCHEDULE 6 AND SCHEDULE 7 SUBSTANCES FOR THE PURPOSES OF EDUCATION, ANALYSIS OR RESEARCH IN TERMS OF THE PROVISIONS OF SECTION 22A(9)(a)(i) OF THE MEDICINES AND RELATED SUBSTANCES ACT, 1965(ACT 101 OF 1965) .

**Date of Issue: 30 January 2025**	**Expiry Date: 29 January 2026**	**Permit No: POS 441/2024/2025**

Authority is hereby granted in terms of Section 22A(9)(a)(i) of the above-mentioned Act to **Prof Gqaleni Nceba** of **University of KwaZulu-Natal, Howard College Campus, Mazisi Kunene Avenue, Glenwood 4041, Durban** to possess and conduct research on plant which is included within schedule 6 to this act. in respect of which the quoted quantity should not be exceeded during the period **30 January 2025** to **29 January 2026.**

Name of Scheduled Substance(s)	Schedule	Total quantity of substance(s) and/or preparation(s) allocated per calendar year
Cannabidiol (CBD)	4	250 ml [two hundred and fifty milliliters]

Total Items: **1**

The acquisition, possess, use or supply of the relevant substances are subject to the following conditions:

1. The substances shall be used for **Research Purposes** only.
2. The control over the substances shall be the responsibility of:

   Full Name & Surname: **Prof Gqaleni Nceba**

   ID Number: **640115 5850 088**
3. Complete details of the substances acquired and used shall be recorded in registers designed specifically for this purpose in accordance with the provisions of the relevant regulations to the Medicines and Related Substance Act, 1965.
4. Other Personnel assisting in Research:

   Full Name & Surname: **Dr Mlungisi Ngcobo**
   Full Name & Surname: **Mr Sanele Mhlungu**
   Full Name & Surname: **Mr Michael Laws**
   Full Name & Surname: **Mr Saziso Ndlovu**
5. When the substances are acquired, the name and address of the supplier, the date supplied, the quantity supplied and the number of the relevant invoice shall be recorded **on this permit**.
6. The register referred to in paragraph 3, as well as copies of orders and invoices pertaining to the supply of the substances, shall be available at the offices of **University of KwaZulu-Natal, Howard College Campus, Mazisi Kunene Avenue, Glenwood 4041, Durban** for a period of at least three years and shall be subject to inspection by Inspectors appointed in terms of the Medicines and Related Substances Act, 1965.
7. This permit expires on **29 January 2025** and shall on expiry be returned to the Department of Health for cancellation and shall be accompanied by a statement reflecting the quantity of substances on stock at expiry.

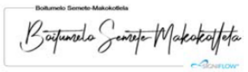

CHIEF EXECUTIVE OFFICER OF SAHPRA:
DR BOITUMELO SEMETE-MAKOKOTLELA
DATE: 31 January 2025

Supplementary Figure S2: Permit issued by the South African Health Products Regulatory Authority (SAHPRA) authorizing the use of medicinal cannabis for research purposes.
